# The Contribution of Prenatal Environment and Genetic Factors to the Association between Birth Weight and Adult Grip Strength

**DOI:** 10.1371/journal.pone.0017955

**Published:** 2011-03-15

**Authors:** Charlotte L. Ridgway, Stephen J. Sharp, Catherine Derom, Gaston Beunen, Robert Fagard, Robert Vlietinck, Ulf Ekelund, Ruth J. F. Loos

**Affiliations:** 1 MRC Epidemiology Unit, Institute of Metabolic Science, Cambridge, United Kingdom; 2 Department of Human Genetics, Katholieke Universiteit Leuven, Leuven, Belgium; 3 Department of Biomedical Kinesiology, Faculty of Kinesiology and Rehabilitation Sciences, Katholieke Universiteit Leuven, Leuven, Belgium; 4 Hypertension and Cardiovascular Rehabilitation Unit, Department of Cardiovascular Diseases, Katholieke Universiteit Leuven, Leuven, Belgium; University of Granada, Spain

## Abstract

Low birth weight has been associated with reduced hand grip strength, which is a marker of future physical function and disease risk. The aim of this study was to apply a twin pair approach, using both ‘individual’ data and ‘within-pair’ differences, to investigate the influence of birth weight on hand grip strength and whether this association may be mediated through fat free mass (FFM). Participants from the East Flanders Prospective Twin Survey were included if born without congenital abnormalities, birth weight >500 g and ≥22 weeks of gestation. Follow up in adulthood (age: 18–34 year), included anthropometric measures and hand grip (n = 783 individuals, n = 326 same-sex twin pairs). Birth weight was positively associated with hand grip strength (β = 2.60 kg, 95% CI 1.52, 3.67, p<0.001) and FFM (β = 4.2, 95% CI 3.16, 5.24, p<0.001), adjusted for gestational age, sex and adult age. Using ‘within-pair’ analyses, the birth weight hand grip association was significant in DZ men only (β = 5.82, 95% CI 0.67, 10.97, p = 0.028), which was attenuated following adjustment for FFM. Within-pair birth weight FFM associations were most pronounced in DZ men (β = 11.20, 95% CI 7.18, 15.22, p<0.001). Our ‘individual’ analyses show that higher birth weight is associated with greater adult hand grip strength, which is mediated through greater adult FFM. The ‘within-pair’ analyses confirm this observation and furthermore show that, particularly in men, genetic factors may in part explain this association, as birth weight differences in DZ men result in greater differences in adult strength and FFM.

## Introduction

The association between low birth weight and increased disease risk in later life is well established [1], [2] suggesting that a compromised foetal environment may have a range of long lasting health consequences. Low birth weight has consistently been associated with reduced hand grip strength in later life [3], [4], [5], [6], [7], which may have important health implications given that muscle strength is a marker of future physical function [8] and disease risk, including cardiovascular disease [9] and all cause mortality [10], [11].

Birth weight is used as a measure of prenatal growth, which is not only determined by the prenatal environment, but also by the foetus' genetic potential. As a consequence, associations between birth weight and adult disease may be due to a compromised environment in utero, or to genetic factors that affect both birth weight and adult disease risk. For example, it has been suggested that the previously observed association between low birth weight and increased risk of type 2 diabetes [2] may be due to genetic variants that are associated with both lower birth weight and increased risk of the disease [12],[13].

Twin studies provide a unique opportunity to disentangle the contribution of foetal growth restriction, maternal environment and genetic factors to the birth weight – adult disease association. This is because both members of a twin pair have the same gestational age and also share the same maternal exposures during pregnancy, such as maternal weight, nutrition and smoking status. However, each individual within a twin pair will have their own foetal-placental environment, which will contribute to differences in birth weight between the two twins. In monozygotic (MZ) twins, these differences in birth weight are solely due to the foetal-placental environment, whereas in dizygotic (DZ) twins differences in birth weight may also be due to genetic as well as environmental differences [14], [15]. Thus by comparing the association, between within-pair difference in birth weight and in adult disease risk, in MZ twins to the association in DZ twins it is possible to estimate the contribution of genetic factors to the birth weight/adult disease relationship.

The aim of this study was therefore to investigate the influence of birth weight on hand grip strength in young adult twins. As it has been hypothesised that the association between birth weight and hand grip may be mediated through fat free mass [7], [16], a secondary aim was to investigate whether this association was independent of fat free mass. Besides the classical cohort analyses, we took advantage of the twin design and examined associations between the within-pair differences in birth weight and within-pair differences in adult hand grip strength or fat free mass, to estimate the contribution of the foetal-placental environment and genetic factors. To the best of our knowledge this is the first study to use within-pair analysis to investigate the birth weight, adult hand grip strength association. We hypothesised that the heaviest twin at birth would have greater adult hand grip strength and increased fat free mass, compared to their lighter brother or sister. By comparing these associations in MZ twins to those observed in DZ twins, we aimed to estimate the contribution of genetic factors and hypothesised that associations would be stronger in dizygotic twins due to genetic differences.

## Methods

### Ethics Statement

The study was approved by the Ethics Committee of the Faculty of Medicine of the Katholieke Universiteit, Leuven, Belgium, and all participants gave written informed consent.

### Study Population

The East Flanders Prospective Twin Survey (EFPTS) is a population-based twin survey that prospectively registered all twins born in the Belgian Province of East Flanders since 1964 [17]. Twins are included if born without congenital abnormalities who meet the World Health Organization criteria for live born infants (birth weight over 500 g and a gestational age of ≥22 weeks) [18]. The EFPTS collects a comprehensive series of markers of early life, including birth weight and gestational age, within 24 h after delivery. Zygosity was determined by sequential analysis based on sex, foetal membranes, umbilical cord blood group, placental alkaline phosphatise and DNA fingerprints [19].

We randomly contacted 803 pairs of all twins born between July 1964 and May 1982 during adulthood, when aged between 18 and 34 years, to participate in the Prenatal Programming Twin Study. Of those, 424 pairs (52.8% overall response rate) agreed to participate [20], [21] and volunteers visited the research centre between February 1997 and April 2000. Data was available for birth weight and adult hand grip for 783 individuals (n = 382 men, n = 401 women).

For the within-pair analyses, we excluded opposite-sex twin pairs (n = 43 twin pairs), as including pairs with discordant sex, where sex may be associated with both birth weight and hand grip, can lead to an overestimation of effect sizes. Pairs of which only one member participated in the study were also excluded, leaving a total of 326 same-sex twin pairs, 170 female pairs (n = 48 female DZ pairs, n = 122 female MZ pairs) and 156 male pairs (n = 43 male DZ pairs, n = 113 male MZ pairs).

### Anthropometric measures

Height was measured barefoot to the nearest 0.1 cm using a wall mounted stadiometer (Harpenden). Weight was measured to the nearest 0.1 kg on a balance scale (Seca, Germany) with the volunteer wearing light clothing. Body mass index (BMI) was calculated as weight (kg)/height^2^ (m^2^). Fat free mass (kg) was estimated using bioelectrical impedance (BIA310, Biodynamics, US).

Hand grip strength of the dominant hand was measured once using a Jamar hand grip dynamometer (Sammons, Preston, UK). Measurements were performed whilst standing, with the arm fully extended next to the body. Hand grip strength was measured on a single occasion after a demonstration and clear explanation by the research associate. Participants were able to familiarise themselves with the hand dynamometer prior to this measurement, without carrying out the test. Once the participants felt ready to carry out the test, they were verbally encouraged by the research associate to perform to their maximum ability.

### Statistical methods

Mean and standard deviation were calculated for descriptive characteristics, for all individuals, for variables collected at birth as well as in adulthood. T-tests were used to investigate differences in means between MZ and DZ twins and between men and women.

Twins were considered as individuals (cohort analyses), to allow comparisons with other cohort studies, and as members of twin pairs (within-pair analyses).

#### Individual Cohort Analysis

Linear regression was performed using mixed effect models, allowing for the fact that twins share more similarities than unrelated individuals, to investigate the associations between birth weight and adult hand grip strength. Similarly, the association between birth weight and adult fat free mass were analysed using linear regression mixed effect models.

Regression models were adjusted for gestational age, adult age and where analysis was carried out in both men and women together the models were adjusted for sex. To investigate whether associations were independent of adult body size, models were additionally adjusted for adult height. Models investigating associations between birth weight and adult hand grip were also adjusted for adult fat free mass to examine whether associations were independent of fat free mass.

Evidence for different effects in men and women were investigated by including an interaction term between birth weight and sex.

#### Within-pair Analysis

The *difference* in birth weight between the first and second born twin is modelled as the ‘exposure’ and the *difference* in hand grip between the first and second born twin is modelled as the ‘outcome’. This allows the influence of birth weight on hand grip strength to be estimated independently from shared maternal influences, such as maternal weight, nutrition and smoking status, and in MZ twins additionally from genetic factors.

Furthermore, comparing the effect size of the within-pair association of MZ twins to those of DZ provides information on the contribution of genetic factors to the birth weight – adult had grip strength relationship. For example, as MZ twin pairs share the same genes, any observed within-pair associations between birth weight and adult hand grip strength are more likely to be due to foetal-placental or environmental influences. Whereas larger effect sizes in DZ twin pairs, compared to MZ twins, would suggest that genetic factors contribute to the association between birth weight and adult hand grip strength.

The associations between within-pair differences in exposure and outcome were analysed using linear regression. The first regression model was presented as unadjusted, or adjusted for sex where analysis was carried out in men and women together. The second regression model included adjustment for the within-pair difference in adult height, to investigate the influence of body size. Finally, hand grip models were also repeated adjusting for sex, within-pair difference in adult height and within-pair difference in adult fat-free mass, to investigate whether these associations were independent of body size and composition.

All analyses were performed using Stata 9 (StataCorp LP, Texas, USA).

## Results

Female twins were just over 106 grams lighter at birth (p = 0.002) and had lower adult hand grip strength (25.6 kg compared to 41.4 kg in men, p<0.001) than male twins (**Table 1**). MZ twins weighed 131 grams less than DZ twins (p<0.001) but there were no differences in adult hand grip strength between MZ and DZ groups (**Table 1**).

**Table 1:** Descriptive statistics of participants stratified by zygosity and gender.

	DZ (n = 302)		MZ (n = 481)		t-test	Men (n = 382)		Women (n = 401)		t-test
	Mean	(SD)	Mean	(SD)		Mean	(SD)	Mean	(SD)	
Birth weight (g)	2621	(480)	2490	(480)	<0.001	2595	(477)	2489	(485)	0.002
Gestational age (weeks)	37.5	(2.4)	36.9	(2.5)	<0.001	37.1	(2.7)	37.5	(2.5)	0.726
Age (years)	26.2	(4.7)	25.2	(4.6)	0.006	25.7	(4.7)	25.5	(4.7)	0.630
Height (cm)	172.4	(8.8)	171.4	(8.9)	0.122	178.3	(6.4)	165.7	(6.2)	<0.001
Weight (kg)	66.6	(12.3)	65.4	(11.3)	0.147	71.0	(10.6)	61.0	(10.6)	<0.001
Adult Body Mass Index (BMI)	22.4	(3.4)	22.2	(3.4)	0.636	22.3	(3.1)	22.7	(3.7)	0.680
Hand grip (kg)	34.0	(9.9)	32.8	(9.8)	0.081	41.4	(7.0)	25.6	(4.6)	<0.001
Fat free mass (kg)	50.9	(9.9)	50.1	(9.4)	0.251	57.9	(6.8)	43.3	(5.6)	<0.001
**Within-Pair differences** *										
Birth weight (g)	51.9	389.7	33.8	335.6	0.649	39.6	353.2	31.4	362.7	0.650
Height (cm)	1.4	5.5	0.1	2.3	0.002	0.4	3.6	0.4	3.4	0.685
Weight (kg)	0.7	10.1	1.2	6.2	0.587	1.0	7.5	1.1	7.7	0.729
Adult Body Mass Index (BMI)	0.2	3.3	0.4	2.1	0.044	0.2	2.5	0.3	2.7	0.469
Hand grip (kg)	0.5	6.1	0.1	5.0	0.458	0.2	5.3	0.3	4.5	0.725
Fat free mass (kg)	0.4	5.8	0.8	3.4	0.460	0.7	4.2	0.6	3.9	0.828

Data are means and standard deviation (SD).

*Within-pair analysis of same sex pairs (men n = 156, women n = 170), (DZ n = 91, MZ n = 235) MZ: Monozygotic twins; DZ: dizygotic twins.

### Individual Cohort Analysis

#### Birth weight and hand grip strength

Birth weight was positively associated with hand grip strength (β = 2.60, 95% CI 1.52, 3.67, p<0.001) with a 1 kg increase in birth weight corresponding to a 2.6 kg increase in adult hand grip strength after adjustment for gestational age, adult age and sex (**Table 2**).

**Table 2:** Associations (β –coefficients; 95% CI) between birth weight and adult hand grip strength or fat-free mass in all individuals.

	All (n = 783)			Men (n = 382)			Women (n = 401)		
	β	(95% C.I)	p	β	(95% C.I)	p	β	(95% C.I)	p
Grip strength (kg) model 1	2.60	1.52, 3.67	<0.001	3.8	2.01, 5.59	<0.001	1.39	0.19, 2.59	0.024
Grip strength (kg) model 2	1.21	0.12, 2.30	0.029	1.95	0.11, 3.79	0.038	0.39	−0.82, 1.60	0.523
Grip strength (kg) model 3	0.96	−0.05, 1.98	0.063	1.36	−0.37, 3.08	0.123	0.40	−0.74, 1.54	0.492
Fat-free mass (kg) model 1	4.20	3.16, 5.24	<0.001	5.86	4.35, 7.37	<0.001	2.89	1.64, 4.14	<0.001
Fat-free mass (kg) model 2	1.68	0.73, 2.63	0.001	2.96	1.48, 4.45	<0.001	0.99	−0.15, 2.12	0.089

Model 1 –model adjusted for gestational age, adult age (and sex when analyses was carried out in men and women together).

Model 2 –model adjusted for gestational age, adult age, (sex) and adult height.

Model 3 – adjusted for gestational age, adult age, (sex) and adult fat free mass.

Linear regression models β coefficients represent change in adult hand grip strength (kg) or adult fat free mass (kg) per 1 kg change in birth weight, with 95% Confidence Intervals.

Additional adjustment for adult height attenuated the strength of association by more than half but remained significant (β = 1.21, 95% CI 0.12, 2.30, p = 0.029). After adjustment for fat free mass the association was further attenuated and no longer significant (**Table 2**).

The strength of association between birth weight and adult hand grip strength in men was more than twice that of women, after adjustment for gestational age and adult age (**Table 2**). However, a formal test for sex interaction was not significant (p = 0.3, data not shown). In men, the strength of the association was halved after further adjustment for height and adjustment for fat free mass further attenuated the association. In women the association was completely abolished after adjustment for either height or fat free mass.

#### Birth weight and fat free mass

We found that higher birth weight was consistently associated with increased fat free mass (β = 4.2, 95% CI 3.16, 5.24, p<0.001) and the strength of this association was more than halved after further adjustment for adult height (β = 1.68, 95% CI 0.73, 2.63, p = 0.001). The association was twice as large in men (β = 5.86, 95% CI 4.35, 7.37, p<0.001) than in women (β = 2.89, 95% CI 1.64, 4.14, p<0.001) when adjusted for gestational age and adult age. Yet, the sex interaction was not significant (p = 0.5, data not shown). Adjustment for height attenuated the association, more so in women than in men (Table 2).

### Within-pair Analysis

#### Birth weight and hand grip strength

The association between within-pair difference in birth weight and the within-pair difference in adult hand grip strength was positive in direction but not significant (β = 1.38, 95% CI −0.27, 3.02, p = 0.101), suggesting that, within a pair, the twin with the higher birth weight tended to have greater adult handgrip strength than their lighter sibling. When stratifying by sex, again there was a positive trend, but this was not statistically significant in either men or women. Further adjustment for intra-pair difference in either height or fat free mass completely abolished the association (**Table 3**).

**Table 3:** Associations (β –coefficients; 95% CI) between within-twin pair difference in birth weight (kg) and the within-twin pair difference in adult hand grip strength or fat free mass.

	All (n = 326 pairs)			Men (n = 156 pairs)			Women (n = 170 pairs)		
	β	(95% C.I)	p	β	(95% C.I)	p	β	(95% C.I)	p
Grip strength (kg) model 1	1.38	−0.27, 3.02	0.101	1.93	−0.91, 4.76	0.182	0.93	−0.96, 2.82	0.332
Grip strength (kg) model 2	0.14	−1.61, 1.90	0.871	0.15	−2.99, 3.29	0.923	0.10	−1.85, 2.05	0.917
Grip strength (kg) model 3	0.10	−1.58, 1.79	0.902	−0.09	−3.17, 2.99	0.954	0.24	−1.59, 2.08	0.796
Fat-free mass (kg) model 1	3.91	2.62, 5.15	<0.001	5.84	3.90, 7.78	<0.001	2.36	0.77, 3.95	0.004
Fat-free mass (kg) model 2	2.23	0.97, 3.48	0.001	4.37	2.25, 6.80	<0.001	0.85	−0.65, 2.35	0.265

Model 1 – unadjusted (adjusted for sex when analyses were carried out in men and women together).

Model 2 – adjusted for (sex) and within twin pair difference in adult height.

Model 3 – adjusted for (sex) and within twin pair difference in adult fat-free mass.

Linear regression models β coefficients represents the difference in adult grip strength (kg) or fat free mass (kg) per 1 kg difference in birth weight between twin 1 and twin 2, with 95% Confidence Intervals.

**Figure 1:**
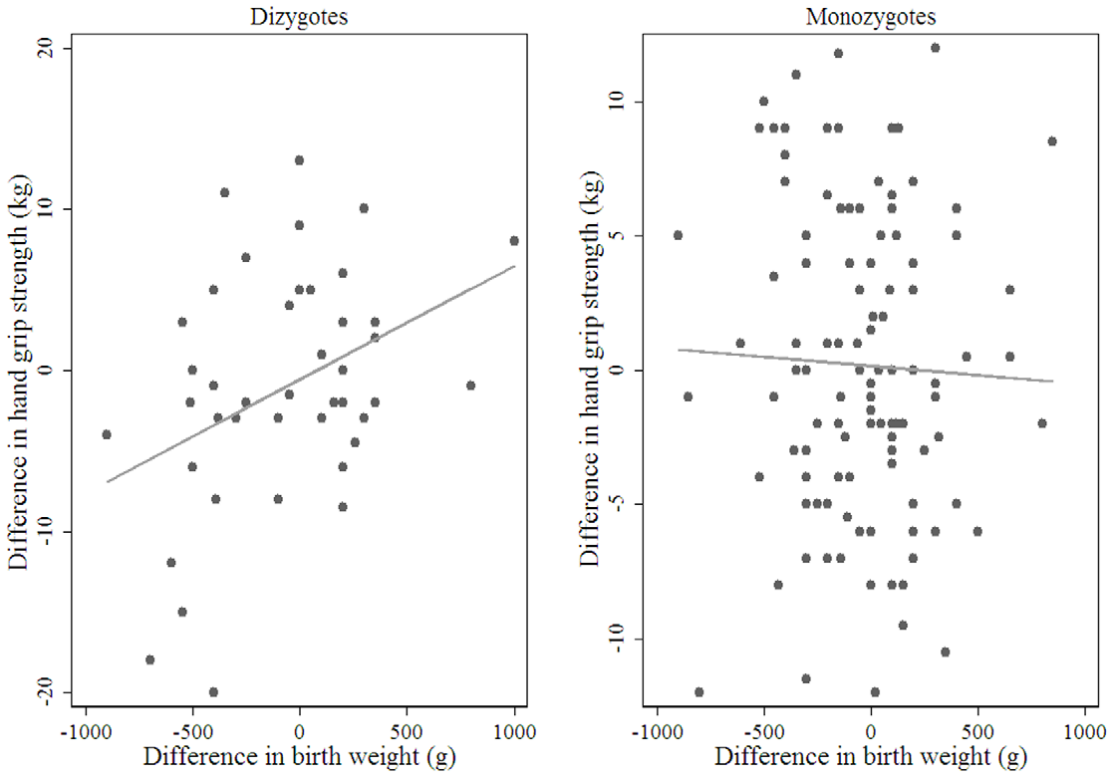
Between pair difference in birth weight and between pair difference in adult hand grip strength in men n = 156 male pairs (n = 43 MZ, n = 113 DZ).

**Figure 2:**
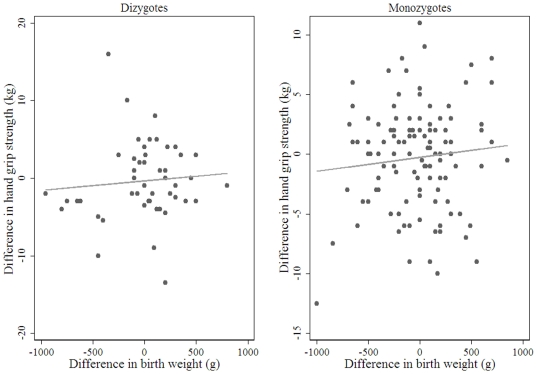
Between pair difference in birth weight and between pair difference in adult hand grip strength in women n = 170 female pairs (n = 48 DZ, n = 122 MZ).

When stratifying by zygosity status, to investigate the influence of genetic factors, we found a strong association between the within-pair difference in birth weight and the within-pair difference in adult hand grip in DZ men, while no association was observed in MZ men (**Table 4** **,** **Figure 1**). The association in DZ men was attenuated following adjustment for within-pair difference in adult height and to a greater extent after adjustment within-pair difference in adult fat free mass (**Table 4**). In women, no associations between the within-pair difference in birth weight and the within-pair difference in adult hand grip of either zygosity was observed (**Table 4**. **Figure 2**).

**Table 4:** Associations (β –coefficients; 95% CI) between within-twin pair difference in birth weight (kg) and the within-twin pair difference in adult hand grip strength or fat free mass stratified by sex and zygosity.

	DZ men (n = 43 pairs)			MZ men (n = 113 pairs)			DZ women (n = 48 pairs)			MZ women (n = 122 pairs)		
	β	(95% C.I)	p	β	(95% C.I)	p	β	(95% C.I)	p	β	(95% C.I)	p
Grip strength (kg) model 1	5.82	0.67, 10.97	0.028	−0.68	−4.09, 2.72	0.692	1.18	−2.92, 5.27	0.565	0.84	−1.30, 2.98	0.439
Grip strength (kg) model 2	3.97	−1.75, 9.70	0.169	−2.12	−6.18, 1.94	0.302	0.86	−3.15, 4.88	0.668	−0.54	−3.04, 1.96	0.668
Grip strength (kg) model 3	2.98	−3.88, 9.85	0.386	−1.57	−5.03, 1.89	0.371	1.97	−2.89, 4.82	0.617	−0.05	−2.20, 2.10	0.964
Fat-free mass (kg) model 1	11.20	7.18, 15.22	<0.001	2.62	0.66, 4.58	0.009	0.57	−3.42, 4.56	0.776	3.02	1.40, 4.64	<0.001
Fat-free mass (kg) model 2	10.21	5.85, 14.57	<0.001	0.65	−1.60, 2.91	0.566	0.00	−3.57, 3.59	0.997	0.51	−1.21, 2.32	0.557

Model 1 – unadjusted.

Model 2 – adjusted for within twin pair difference in adult height.

Model 3 – adjusted for within twin pair difference in adult fat-free mass.

Linear regression β coefficients represents the difference in adult grip strength (kg) or fat free mass (kg) per 1 kg difference in birth weight between twin 1 and twin 2 with 95% Confidence Intervals (MZ Monozygotic twins; DZ: dizygotic twins).

#### Birth weight and fat free mass

Consistent with the cohort analyses, we observed a positive association between the within-pair difference in birth weight and the within-pair difference in fat free mass, indicating that the higher birth weight twin had more fat free mass in adult life compared to their lower birth weight twin. Again, this association was more pronounced in men than in women. Adjustment for height attenuated the association in men, and completely abolished the association in women.

Positive associations between the within-pair difference in birth weight and the within-pair difference in adult fat free mass were observed in both DZ and MZ men, but this association was of a more than 4-fold greater magnitude in DZ men compared to MZ men (**Table 4**) and a formal test for interaction between birth weight difference and zygosity was significant (p = 0.027, data not shown). Following further adjustment for the within-pair difference in adult height, the association in DZ men was only slightly attenuated, but was abolished in MZ men.

The opposite patterns of association were observed in women, as an association between the within-pair difference in birth weight and the within-pair difference in fat free mass, was only observed in MZ women but not in DZ women (**Table 4**), although the differences between MZ and DZ was very small and there was no evidence for a birth weight difference * zygosity interaction (p = 0.88, data not shown). However, no associations were observed in either MZ or DZ women after adjustment for the within-pair difference in adult height.

## Discussion

Our cohort analyses that consider twins as individuals show that a higher birth weight is associated with greater adult hand grip strength, which is predominantly mediated through the association between birth weight and fat free mass. The overall trend from the twin pair analyses, which controls for shared factors, confirms the cohort observations and suggests that within a pair the heavier twin at birth has greater hand grip strength in adult life than their lighter sibling. Uniquely, the twin pair analysis identified that the association between birth weight and hand grip strength was stronger in DZ than in MZ men, suggesting a potential influence of genetic factors, acting on both on birth weight and adult hand grip in men.

The positive associations between birth weight and adult hand grip strength are consistent with those observed in singletons and show similar effect sizes [3], [4], [5], [6], [7]. Also the positive associations between birth weight and fat free mass are also consistent with previous studies in singletons, for both children [22], [23], [24], [25] and older adults [26], [27]. Furthermore, the magnitude of the association between birth weight and adult hand grip in women was around half of that seen in men, again consistent with previous studies [4], [6], [7].

Our findings suggest different patterns in the association and mediation between birth weight, and hand grip and fat free mass for men and women. In women the association between birth weight and hand grip strength was completely abolished after adjustment for adult height or fat free mass. In addition the association between birth weight and adult fat free mass in women was substantially reduced after adjustment for adult height. In men, however, while the association with hand grip is attenuated after adjustment for adult height or fat free mass, there remains a residual effect of birth weight on adult fat free mass independent of adult height. This suggests that the association between birth weight and grip strength is mediated through both adult height and fat free mass in women, whereas in men the association is largely mediated through fat free mass.

The gender-specific association between birth weight and adult fat free mass is consistent with the results observed in a study in children that used the more sensitive four-compartment measurement of body composition. In this study, birth weight was associated with height in both boys and girls, but birth weight was only associated with fat free mass (measured as Fat Free Mass Index = FFM/height^2^) in boys [22]. However, the evidence for gender-specific differences in the birth weight and fat free mass association is not consistent across other studies in children and adolescents. Some studies have only observed an association between birth weight and fat free mass in females [28], [29], while others have not observed gender differences in the association between birth weight and later fat free mass [23], [25].

A potential explanation for the difference observed in the birth weight fat free mass association observed in this study, which was only independent of height in men, is that adult muscle mass distribution is known to vary between men and women [30]. Men have proportionally more upper body muscle mass and women more lower body muscle mass [31], and adjustment for height tends to explain more of the variation in lower body muscle mass [32].

A novel finding in this study was the significant association, using within-pair analysis, between birth weight and adult hand grip strength in DZ but not in MZ men. This suggests that, at least in men, the association between birth weight and adult grip strength may in part be explained by genetic influences that act on *both* birth weight and adult hand grip. A similar pattern is observed for fat free mass; i.e. in the association was more pronounced in DZ men than in MZ men, again suggesting that while in utero growth restriction influences adult fat free mass, there are additional genetic factors acting on both birth weight and later fat free mass.

There are however some limitations to this present study. While the twin pair analysis has a number of advantages for disentangling genetic and in utero effects, it has typically less statistical power to detect associations as compared to the individual cohort analyses. Firstly, because using twin pairs as the unit of analysis effectively halves the sample size, secondly the within-pair analysis uses the *difference* in birth weight between the first and second born twin. The range of such within-pair differences are typically much smaller than the range of birth weights observed for individuals using conventional cohort analysis, making it harder to detect associations, so it is possible that the within-pair analysis was underpowered. Furthermore, it should be noted that this present study used bio-electrical impedance to estimate fat-free mass, which may not be as accurate as other laboratory based measures such as duel x-ray absorbtometry, densitometry or deuterium dilution, although many of these more detailed measures are less feasible in larger studies. It should also be noted that we did not measure physical activity, which can influence both fat-free mass and physical performance. Therefore, it is possible that the associations between birth weight and fat-free mass may be mediated by physical activity levels. Finally, we acknowledge that twins may not be representative of singletons, as twins tend to be born both earlier and lighter. Therefore, the results should be interpreted with caution. However, we should highlight that our twin sample is randomly drawn from a prospective, population-based cohort with detailed information collected at birth and with objective measures of muscle strength and body composition in young adulthood.

Future large scale twin studies would be particularly useful to investigate the developmental origins of physical performance and body composition further. Elucidating the influences of shared maternal factors, both in terms of biological influences such as maternal weight and nutrition status, as well as confounding from socio-economic factors, is one of the key issues when investigating the developmental origins of health and disease. Therefore, future twin studies with detailed maternal measures of these variables would be particularly useful.

Future genome wide association studies may identify common genetic variants influencing birth weight, muscle strength and body composition. For preventive purposes, it would be particularly interesting to understand whether the associations between these potential gene variants with future body composition and physical function, including muscle strength, are modified by lifestyle factors, such as increased physical activity.

In summary, our findings suggest that birth weight is positively associated with adult hand grip strength, which appears to be mediated predominantly via influences of birth weight on fat free mass. Furthermore, in men these associations may, at least in part, be influenced by genetic factors that act on both birth weight and later hand grip strength and fat free mass. These findings suggest that a compromised early life, due to either growth restriction in utero or other maternal factors, may be important for both hand grip strength and fat free mass in adulthood, which has implications for both physical function and disease risk in later life.
